# Phosphor-Free InGaN White Light Emitting Diodes Using Flip-Chip Technology

**DOI:** 10.3390/ma10040432

**Published:** 2017-04-20

**Authors:** Ying-Chang Li, Liann-Be Chang, Hou-Jen Chen, Chia-Yi Yen, Ke-Wei Pan, Bohr-Ran Huang, Wen-Yu Kuo, Lee Chow, Dan Zhou, Ewa Popko

**Affiliations:** 1 Graduate Institute of Electro-Optical Engineering, Chang Gung University, Taoyuan 333, Taiwan; davenlee15@gmail.com (Y.-C.L.); os0110742@gmail.com (H.-J.C.); iamalc0405@hotmail.com (C.-Y.Y.); saintsmallpan@yahoo.com.tw (K.-W.P.); 2 Department of Otolaryngology-Head and Neck Surgery, Chang Gung Memorial Hospital, Taoyuan 333, Taiwan; 3 Department of Materials Engineering, Ming Chi University of Technology, New Taipei City 243, Taiwan; 4 Graduate Institute of Electro-Optical Engineering, National Taiwan University of Science and Technology, Taipei 10607, Taiwan; huangbr@mail.ntust.edu.tw (B.-R.H.); merlin955063@hotmail.com (W.-Y.K.); 5 Department of Physics, University of Central Florida, Orlando, FL 32816, USA; lee.chow@ucf.edu; 6 Department of Materials Science and Engineering, University of Central Florida, Orlando, FL 32816, USA; dzhou@pegasus.cc.ucf.edu; 7 Institute of Physics, Wroclaw University of Technology, 50-370 Wroclaw, Poland; Ewa.Popko@pwr.edu.pl

**Keywords:** InGaN/GaN, quantum dot, quantum well, phosphor free, white LED

## Abstract

Monolithic phosphor-free two-color gallium nitride (GaN)-based white light emitting diodes (LED) have the potential to replace current phosphor-based GaN white LEDs due to their low cost and long life cycle. Unfortunately, the growth of high indium content indium gallium nitride (InGaN)/GaN quantum dot and reported LED’s color rendering index (CRI) are still problematic. Here, we use flip-chip technology to fabricate an upside down monolithic two-color phosphor-free LED with four grown layers of high indium quantum dots on top of the three grown layers of lower indium quantum wells separated by a GaN tunneling barrier layer. The photoluminescence (PL) and electroluminescence (EL) spectra of this white LED reveal a broad spectrum ranging from 475 to 675 nm which is close to an ideal white-light source. The corresponding color temperature and color rendering index (CRI) of the fabricated white LED, operated at 350, 500, and 750 mA, are comparable to that of the conventional phosphor-based LEDs. Insights of the epitaxial structure and the transport mechanism were revealed through the TEM and temperature dependent PL and EL measurements. Our results show true potential in the Epi-ready GaN white LEDs for future solid state lighting applications.

## 1. Introduction

In nitride based semiconductors, the indium gallium nitride (InGaN) compound semiconductor has been used to fabricate light emitting diodes (LEDs) and laser diodes ranging from ultra violet to visible light by modulating the composition of indium-gallium ratio to vary the correspondent energy gap between 0.7 eV and 3.4 eV [1,2,3,4,5]. Except the output power, an ideal white light illumination for in-door lighting requires the light source to have the Commission International de l’éclairage (CIE) coordinates, which are similar to that of a blackbody radiator with correlated color temperature (CCT) in the range of 2500–6500 K, and with a color-rendering index (CRI) above 70–80 [6,7]. In 1996, Nichia Corp. developed the first white light emitting diodes (LEDs) using the GaN based blue LEDs and yellow yttrium aluminum garnet (YAG) phosphor. At present, most of the mature white LEDs are still based on the blue InGaN chips coupled with the yellow-emitting YAG phosphor. Although this technology has been widely adopted in commercial white LEDs, extra costs have resulted from the high reaction temperature (over 1800 °C) and the high pressure required for synthesizing phosphors, which is a critical drawback. Many other sulfides and fluoride phosphors [8,9] have also been considered, however, poor moisture resistance and the drop in light intensity of these materials limit their practical applications. To date, sustaining a constant luminous efficiency and CRI value of the phosphor-based white LEDs (WLEDs) are still major objectives for the LED industry. Many research groups aimed to develop a gallium nitride (GaN)-based phosphor-free white LED utilizing two-color InGaN quantum structures, which we discuss below.

In 2001, Damilano et al. [10] demonstrated molecular beam epitaxy (MBE) fabricated monolithic, phosphor-free, white LED wafers with a stack of InGaN/GaN quantum structures, and their low output power electroluminescence (EL) emitted dual wavelengths for the first time. Since then, various groups have fabricated monolithic phosphor free white LEDs [11,12,13]. For example, Huang et al. [11] used a pre-strained metal organic chemical vapor deposition (MOCVD) technique to grow yellow emitting InGaN/GaN quantum structures with increasing Indium concentrations, then, by adding a blue emitting quantum well (QW) at the top of the pre-strained layer, they were able to fabricate monolithic white LEDs with a color temperature of 5600 K by on-wafer probing. Later, Liu et al. [12] also fabricated dual wavelength InGaN/GaN multiple quantum well-wafers by the MOCVD method. However, they only reported their photoluminescence (PL) properties at high indium concentrations, and the internal quantum efficiency drops rapidly. Recently, Chi et al. [13] demonstrated a GaN-based two color LED with a p-type insertion layer to control the intensity ratio of two-color emission and also prevented the inter transition. However, despite all the research efforts, precise process control is still needed to extract the light out of the blue quantum layer, and this is a problem for the practical commercial application. We should mention briefly here that, recently, Zetian Mi et al. demonstrated a high output power phosphor-free nanowire WLED with an unprecedentedly high CRI of 92–98 [14] and then successfully enhanced the electrical and optical properties by fabricating a phosphor-free tunnel junction nanowire WLED [15,16]. That is a topic for another time.

In the field of industrial compound semiconductor epitaxial growth, the lattice mismatch between different growth layers with different composite materials can lead to strain or stress, and the release of this strain or stress can cause phase separation [17,18]. In recent years, lattice relaxation has been used to control the precipitation of indium in the GaN/InGaN quantum wells, thereby enabling the emitted light to change from green to a longer wavelength, or even to produce indium-rich clusters of epitaxial wafers [19]. These quantum dots exist in the GaN/InGaN QW interfaces [20]. For this reason, many attempts have been made recently to replace the phosphor-based LEDs [21,22] with the phosphor-free white LEDs [23]. It is known that the blue band gap quantum wells are transparent to the green-yellow light emitted from the green-yellow quantum dots (QDs). Thus, the green-yellow QD layers should be the first epitaxial layer (on the bottom), followed by the blue QW layer (on the top). Unfortunately, this tandem structure is very difficult to grow at present, since the high indium composition QD layers encounter many problems such as phase separation, dislocation, and extended defects. These problems will prevent the high quality epitaxial growth of the blue QW layer and will cause the completed two-color tandem structure to have a poor light emitting efficiency. This is attributed to the dislocations and defects induced by the large lattice mismatch between the lattices of indium rich In_x_Ga_1-x_N and GaN, which act as non-radiative recombination centers, and, thus, weaken the internal quantum efficiency [24]. These obstacles are often encountered in the production of QD/QW white LEDs and have resulted in a few reports in the literature on the GaN-based phosphor-free white LEDs. Furthermore, the low CRI value during high current operation is another serious problem for the GaN-based phosphor-free white LED. At high current operation, the n-side QD ground state transition will be saturated at first, and the blue QW ground state transition, as well as the blue QW to green-yellow QD tunneling transition, will increase. In the end, the blue light emission will dominate the output spectrum and will cause a low CRI value.

**Figure 1:**
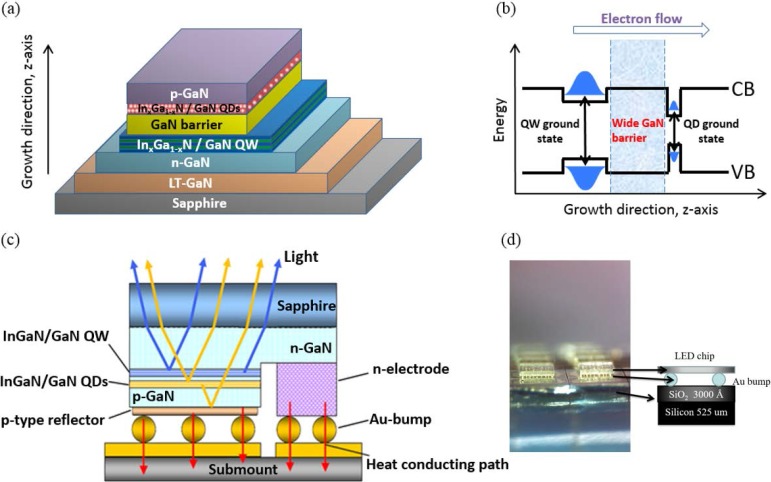
(**a**) The schematic diagram of the white light emitting diode (LED) samples based on indium gallium nitride (InGaN) QDs and QWs; (**b**) A thick tunneling block layer was inserted between QD and QW layers; (**c**) The schematic diagram of the QD/QW white LEDs with flip-chip mounting; (**d**) The picture of fabricated QD/QW white LED after the flip chip (FC) process.

In this work, to realize the high CRI phosphor-free white LEDs, a tandem structure is proposed. It includes low indium ratio blue QW layers on the bottom, high indium ratio green-yellow QD layers on the top to improve epitaxial growth, together with a thick tunnel barrier (TB) layer to prevent the QW-QD coupling transitions; see Figure 1a,b. After the epitaxial growth, the phosphor-free WLED was processed and fabricated into chips. We applied a thermal sonic flip chip (FC) bonding technology, as shown in Figure 1c,d, to bond the fabricated phosphor-free white LED chip to a silicon sub-mount [25,26,27,28,29], which acted as a heat sink. After the FC processing, the green-yellow QD is on the bottom, the blue QW is the top, and the emitted green-yellow light from the QD can easily penetrate the top blue QW layers and produce a high CRI value of 82, even under the elevated current operation conditions of 350–700 mA. Current-voltage (I–V) measurements, electroluminescent sphere integration measurements, and temperature variation photoluminescence spectroscopy have been carried out to investigate the correspondent optical and electrical properties of the fabricated phosphor-free QW/QD white LED in the following text.

## 2. Experiments

The proposed two-color phosphor-free white light epitaxial wafer with a thick TB layer used for fabricating phosphor-free white LEDs were grown at A-STAR Singapore using a metal organic chemical vapor deposition system (MOCVD). The technologies of high-efficiency InGaN/GaN QW-based blue LEDs are quite mature and these LEDs are commercially available [30]. However, many arduous challenges to improve the performance of the green-yellow LEDs still exist [31]. The most difficult part is the large lattice mismatch (11%) between the GaN and indium nitride (InN). The increase in indium content becomes difficult owing to the miscibility among these binary or ternary compounds. Therefore, in this work, we start with the epitaxial growth of QW first, then grow the thick TB layer, and finally deposit the high indium ratio green-yellow light emitting QD layers [32].

The LED structure layers were grown on a C-oriented (0001) 2-inch-diameter sapphire substrate. After the thermal annealing process, all samples consisted of a 25 nm GaN buffer, 1.2 μm undoped GaN, and 1.5 μm graded doped n-GaN layer. Then, three layers of QW, a 11.8 nm TB layer, and four layers of green-yellow QD structure were deposited. Here, the QW layers consisted of In_0.19_Ga_0.81_N (blue) and In_0.23_Ga_0.77_N (green) structures and the QD layers consist of different aggregate sizes. To prevent the continuing growth of the green-yellow QW/QD layer and blue QW layer that was seen previously [33], we have grown a thick 11.8 nm block layer in between the blue QW and green-yellow QW/QD layers to prevent inter-transition. Finally, a thick p GaN layer was grown on top of the QD/QW grown layer. After the epitaxial growth, a low temperature thermal activation process was performed. Then, a chip with dimensions of 1 × 1 mm^2^ was fabricated by the standard process of lateral contact electrodes. A 300-nm-thick indium-tin-oxide (ITO) film, used as the current spreading layer, was deposited by electron-beam evaporation, and the Cr/Pt/Au metal films were used as the n- and p bonding pad contacts. These white LED processes and the subsequent FC package processes were carried out in the LED fabrication facility at the Green Technology Research Center of Chang Gung University. We used the FC technology with gold bumps as the heat dissipation path to enhance both the light mixing property and the cooling capacity of the fabricated QD/QW white LED [29].

The TEM samples were prepared by using the focused ion beam (FIB, FEI FIB200) system with a 30 kV Gallium liquid metal ion source and the scanning transmission electron microscope (STEM)/TEM analysis was carried out by employing the FEI Tecnai F30 TEM with a field emission electron filament operated at 300 kV. Both FIB and STEM works were carried out at the University of Central Florida.

For the PL temperature dependent experiments, the sample was placed in a continuous flow liquid helium cryostat and experiments were performed by using a HeCd laser with a wavelength of 325 nm. The EL in the temperature investigation was detected using a photometric integrating sphere. The samples were mounted on a substrate of aluminum-copper alloy, and then fixed on the hotplate by using thermos paste. The thermocouple probe was pasted on the edge of the LED module for maintaining the temperature from 50 to 170 °C.

## 3. Results and Discussion

### 3.1. Characterizations of the QD/QW Structure

**Figure 2:**
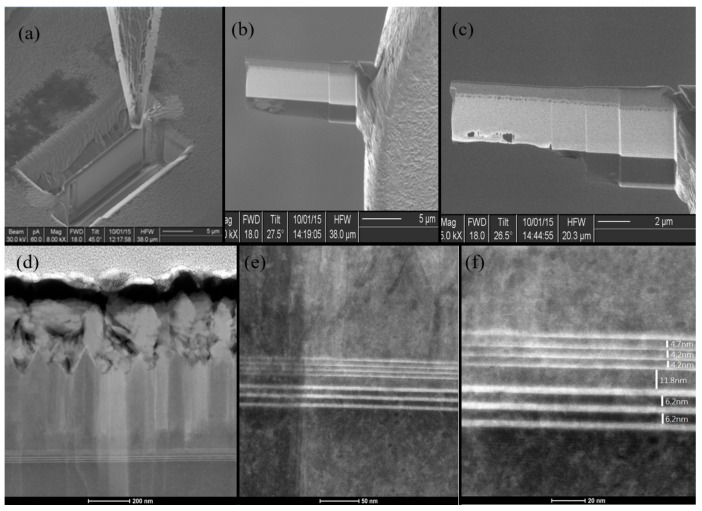
(**a**) FIB-TEM sample preparation, selective area, and deposit Pt bar; (**b**) After FIB etching, using a W needle to lift out the section; (**c**) Thin FIB sample section mounted on a TEM sample grid and further thinning and polishing of the processed section; (**d**) An overall STEM image at low magnification, QD/QW structures are about 800 nm deep to the surface; (**e**,**f**) The STEM images at high magnification with four QD structures on top and three QW layers at the bottom are clearly observed.

After the epitaxial growth, to acquire an overall image of the grown structure, the TEM sample was prepared by the FIB, and images are shown in Figure 2. Before the FIB-TEM sample preparation, the Pt bar was deposited as an ion mask, as shown in Figure 2a. After the FIB ion etching, we used a tungsten needle to lift out the selected section from the epitaxial growth wafer, as show in Figure 2b. Next, the thin FIB sample section was mounted on a TEM grid, then the final step was to further thin and polish the selected section, as shown in Figure 2c.

A STEM image at low magnification was obtained, and the corresponding growth of the QD/QW were about 800 nm below the top surface, as shown in Figure 2d. The STEM images revealed four layers of the green-yellow QD structure on top of the three layers of the blue QW structure, which are shown in Figure 2e,f with different magnifications. As we demonstrated in Figure 1a, the low indium ratio blue QW layers were grown first and then were followed by the high indium green-yellow QD layers, which makes the epitaxial growth of this tandem structure much easier than the opposite tandem structure [34]. Measured from the TEM images, the thickness of the barriers between the QDs and the QWs were about 4.4 and 6.2 nm, respectively. As we proposed, a 11.8 nm TB layer was grown in between the QD and the QW layers to prevent coupling transitions from the QW to the QD [35]. In our previous study, the InGaN/GaN superlattices were helpful to improve the indium composition in the InGaN QDs [5].

**Figure 3:**
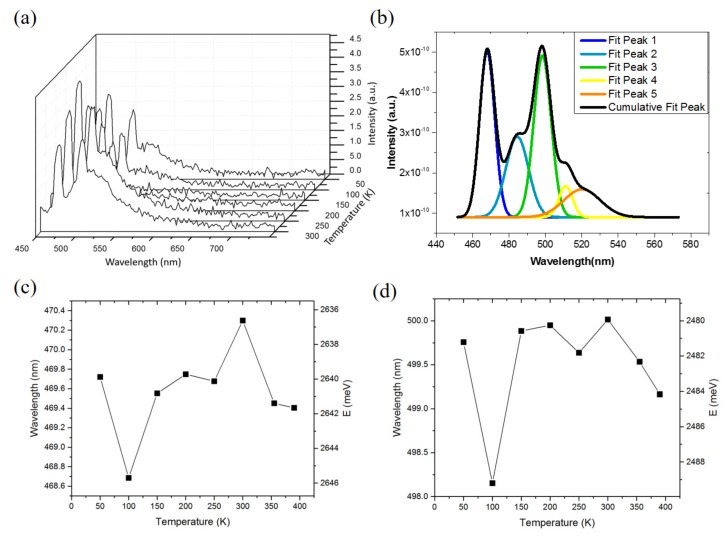
(**a**) The temperature dependent photoluminescence (PL) spectra from 50 to 300 K; (**b**) The PL spectrum of a grown QD/QW wafer at temperature 20 K, and the decomposed sub peaks from the PL spectrum by using a software Origin 8.1; (**c**) the PL peak 1 spectra of the cool white QD LEDs with varied temperature from 50 to 400 K; (**d**) the PL peak 3 spectra of the cool white QD LEDs with varied temperature from 50 to 400 K.

Figure 3a shows the temperature dependent PL spectra from 50 to 300 K. Figure 3b presents the PL spectrum of a QD/QW wafer at a temperature 20 K and the side peak which was decomposed into several sub peaks. The incongruency of the observed peaks in Figure 3a and the sub peaks in Figure 3b are attributed to the non-uniform epitaxial growth of the QDs. Figure 3c,d summarizes the temperature dependency wavelengths of PL peaks 1 and peak 3, respectively. The total shifts of the PL peaks 1 and 3 from 50 to 400 K are 2 and 3 meV, respectively. These shifts are all less than 5 meV, while those shifts in the InGaN bulk are more than 30 meV, as reported in the literature [36]. In a previous report of the PL properties of the InGaN/GaN QDs [37], as the temperature increases, carriers can be thermally transferred into low-energy states from high-energy states. The small shift observed in our samples may be due to a similar effect.

### 3.2. Device Characterization of QD/QW LEDs

**Figure 4:**
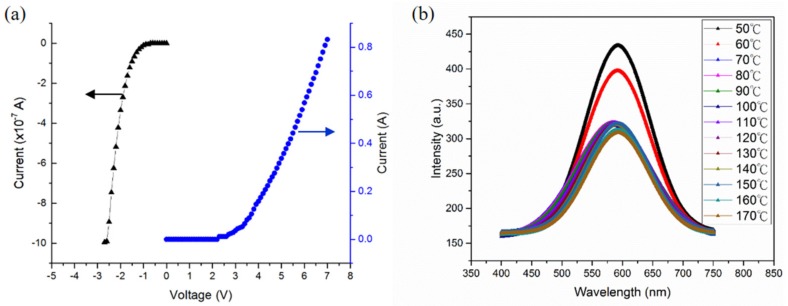

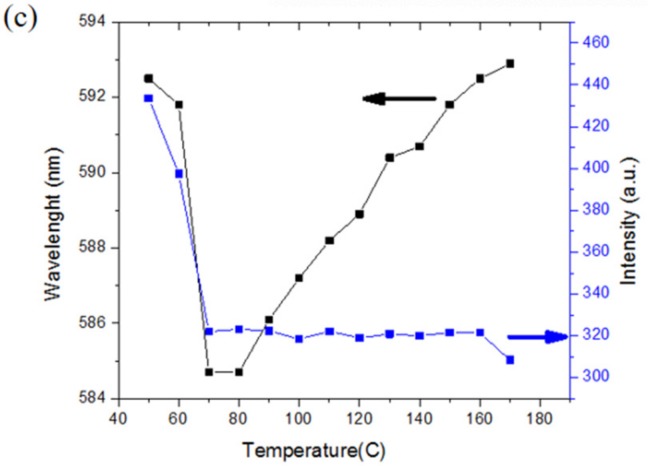
(**a**) The current voltage (IV) curves of the white QD LEDs at room temperature; (**b**) The temperature dependent electroluminescence (EL) spectra of the white QD LEDs at 50 °C–170 °C.; (**c**) The temperature dependent EL peak intensity and wavelength at 350 mA.

Figure 4a shows the room temperature current voltage (IV) curves. At the forward bias, the turn-on voltage of the fabricated QD/QW WLED is approximately 2.8 V, with a standard injected current of 350 mA. The slope of its IV curve is larger than the commercial ones, which implies an excessive series resistance. This excess resistance is thought to be due to the stack structure itself, which includes a high concentration of grown defects accompanied with the QD formation process and the extra FC packaging process as well. After close examination of the leakage current at the reverse bias, it was revealed that the leakage current of the QD/QW WLED under a reverse bias of −2.5 V was almost 1 μA and was thought to be due to the reverse tunneling current from the high concentration defects of the Indium (In) rich grown layers [38,39].

In efficient, QW, LED electroluminescence, the majority of the injected electrons and holes should be converted into photons through radiative recombination. However, in most QD LED works, they were converted into phonons via non-radiative recombination and are eventually transferred to heat. This is the reason why a direct observation of the EL emission in the GaN based QD/QW white LED is difficult. In this work, Figure 4b shows the temperature dependent EL spectra of the white QD LEDs under 350 mA injection current from 50 °C to 170 °C, and Figure 4c successfully displays the temperature dependent EL spectra from 50 °C to 170 °C, respectively. In this figure, the broad EL spectra, with the wavelength extending from 475 nm to 675 nm, and a broad peak centered at 570 nm are shown, which is close to the ideal wavelength range of a white-light source. However, some noise peaks were occasionally found during the EL measurement for devices picked from the same growth wafer, which indicates that the fabricated white LEDs exhibited not only the lattice mismatch induced defects, but also the non-uniform QD distributions which are also consistent with the large leakage current phenomenon of IV curves.

As the temperature increases, a decrease of their intensity is due to both the carrier overflow and the vibrations in the lattice as the temperature rises, which decrease the probability of a radiative recombination and increases the probability of a non-radiative recombination. At the QD/QW white LEDs’ EL spectra, the changes in the peak wavelength can be categorized in terms of the two temperature zones. The first zone lies between 50 °C and 70 °C, where a blue shift of the main peak from 593 nm to 585 nm takes place. This result may be attributed to the suppression of the quantum confinement Stark effect [40]. The second zone lies between 70 °C and 170 °C, where the main peak displays a steady red shift from 585 nm to 593 nm which is caused by the lattice expansion and vibration [41]. Nevertheless, a low power output was determined from the EL measurements, which can also be attributed to the inferior crystalline quality of the QD, due to its highly dense and unfavorable composition in the InGaN QD layers, and a further study of the epitaxial growth conditions is needed [33,42].

### 3.3. Light Mixing Properties

**Figure 5:**
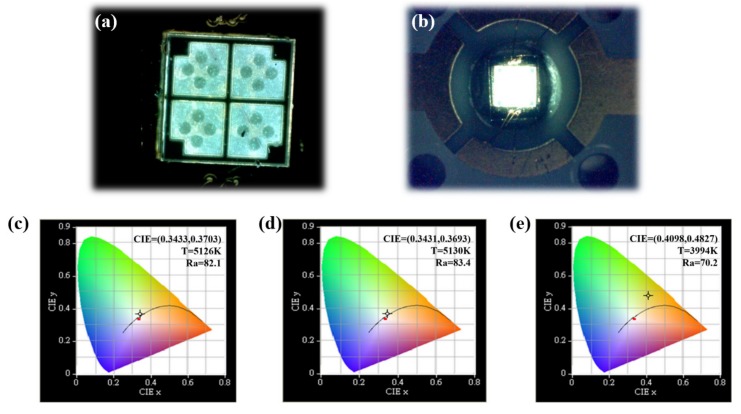
(**a**) The images of the FC package QD/QW cool white LED driven at 5 mA; (**b**) The images of the light outputs from QD/QW white LEDs were taken as the current increased to 50 mA. The color temperatures and color coordinates of color rendering of white QD/QW LEDs at different bias currents of (**c**) 350 mA; (**d**) 500 mA; and (**e**) 700 mA.

After the FC package, the FC devices were checked by a probe station with a bias current of 50 mA to obtain a clear picture of these devices with bonding pads. The QD/QW white LED is shown in Figure 5a. The blue, cold, white output light was obtained, which is due to the fact that the electron has a higher mobility than a hole, and it dominates the light sequence, which is injected from the bottom n-side to the low indium QW layer to emit a blue light first. Thus, a short wavelength blue light emits through the sapphire substrate. As the bias current increased to 350 mA, the injected electrons go deeper into the green-yellow QD layer and the output becomes brighter, as shown in Figure 5b. Finally, at 500 mA, the long wavelength QD layer emits a green-yellow light mixed with a blue light, forming a warm white light as shown in Figure 5c. By mixing the emission lights from the QDs and QWs, the fabricated QD/QW white LED emits a warm white light, as the photos show in Figure 5c. We applied various bias currents of 350 mA, 500 mA, and 700 mA to the FC QD/QW white LED, and used an integrating sphere to measure its correspondent color temperature and CRI.

Table 1 presents the luminous efficacy of the QD-LED at the different bias currents. At its peak, the luminous efficacy was about 0.0024 lm/W at 350 mA, which is considerably lower than the value 70~100 lm/W of common LEDs.

Figure 5d–f demonstrates the QD/QW white LEDs’ chromaticity diagrams of the color space resulting from the different bias currents of 350, 500 and 700 mA, respectively. At the QW/QD tandem system, coupling probability between the QW ground state and the QD ground state is inversely proportionate to the thickness of the tunneling block layer. Thus, in our design, a 12 nm TB layer was inserted in between the QD and QW layers to prevent the tunneling transition taking place, which makes the emitted blue light dominate and decreases the CRI value considerably [33]. As shown in Figure 5d,f, QD white LED’s color temperature and the CRI at the bias currents of 350 and 500 mA reached 5126 and 5130 K, 82 and 83.4, respectively. These values are good enough in comparison to the conventional phosphor-based LED, which has a color temperature of 4500 K and a CRI value of 75. In addition, these values are much better than a recent report with a CRI value as low as 13, in which a reverse tandem QW/QD white LED structure without a TB layer had been fabricated [33]. Here, we report the color temperature and the CRI still have good values of 3994 K and 70, respectively, even as the bias current is increased to a value as high as 700 mA.

In the current dependent thermal measurement, we observed that under a 350 mA operation, the junction temperature of our WLED is approximately 120 °C, whereas at the 700 mA operation, the junction temperature reaches as high as 200 °C. Table A1 presents the CRI test of the white LED according to DIN 6169 at the 350, 500 and 700 mA operation, respectively. The corresponding CRI decreased from 82 to around 70, when the current increased from 350 mA to 700 mA because the red light performance index of R9 dropped from 40 to 35. The reduced red band observed at 200 °C also contributed to the blue shift.

## 4. Conclusions

The fabrication of a phosphor-free long-wavelength-top tandem cell QD/QW cool white LED was accomplished by MOCVD epitaxial growth followed by flip-chip technology. Because FC technology is applied, the grown sequence can be arranged with the low Indium (In) ratio layers first and the high In ratio layers second, which makes the epitaxial process easier. That also means the 4-layers of In-rich QD structure are grown on top of the 3-layers of low In blue QW structure. After the flip chip process, the conventional epitaxial growth that results in a blue light absorption problem in the high In ratio layer was then solved. In addition, with conventional QD/QW tandem structures, those layers are grown continuously with a thin block layer or without a block layer which results in a low CRI during high current operation. In this work, the QD/QW inter transition problem was solved by inserting a 11.8 nm TB layer. The successful EL exhibited a distribution of the emission spectrum that varied from 450 to 700 nm, which is very close to the ideal white-light source and is wider than that of the phosphor-based white LEDs. At a standard bias current of 350 mA, a color temperature of 5126 K, and a CRI value of 82.17, are presented, which is better than the recently reported results with a reverse epitaxial structure.

However, those single-chip QW/QD cool white LEDs have an unexpected drawback of low luminous efficiency. The requirement of overall luminous efficiency of QW/QD LEDs can be improved by optimizing the thickness, growth conditions, and the concentration of dopant in the QW and QD layers. Furthermore, the thickness of the TB layer might have reduced the efficiency more than expected due to its increased resistance. These factors present an important basis for the fabrication of single-chip white LEDs. Thus, an efficient phosphor-free white InGaN based LED requires more time and research.

## Figures and Tables

**Table 1:** Luminous efficacy of white QD-LED at 350, 500 and 700 mA. CIE stands for Commission International de l’éclairage.

Set Current Value (mA)	Relative Color Temperature (K)	CIE *x*	CIE *y*	Wavelength (nm)	Color Purity (%)
350	5126	0.34	0.37	564.3	14.17
500	5130	0.34	0.36	564.36	13.81
700	3994	0.40	0.48	570.25	67.99

## Appendix A

**Table A1:** The 14 CRI test colors of FC package QD/QW WLED according to DIN 6169 at the bias currents of 350, 500 and 700 mA.

Bias Current	350 mA	500 mA	700 mA
R1	92.41866	88.78911	60.93052
R2	92.13289	95.05205	71.70682
R3	77.45595	79.1353	87.9434
R4	71.69764	73.43228	66.92189
R5	88.66449	87.51749	61.88037
R6	90.6693	94.98337	65.34932
R7	75.32654	78.32166	91.69814
R8	69.06432	70.10013	55.46916
R9	43.20995	40.38482	−35.15676
R10	81.89597	87.42805	40.71531
R11	75.54762	77.11202	56.45898
R12	77.31905	74.24747	43.0356
R13	95.75813	93.01258	61.30183
R14	87.0627	87.52311	94.5731
Ra	82.17872	83.41642	70.23745
